# Supplementary material for: The 3′ Pol II pausing at replication-dependent histone genes is regulated by Mediator through Cajal bodies’ association with histone locus bodies
Source: Nat Commun. 2022 May 25;13:2905. doi: 10.1038/s41467-022-30632-w (PMC9133132; doi:10.1038/s41467-022-30632-w)
Supplement: Supplementary file 1 — Supplementary Information [file 41467_2022_30632_MOESM1_ESM.pdf]

Supplemental Information, Suzuki et al.

The 3′ Pol II pausing at replication-dependent histone genes is regulated by Mediator through Cajal bodies' association with histone locus bodies

Hidefumi Suzuki, Ryota Abe, Miho Shimada, Tomonori Hirose, Hiroko Hirose, Keisuke Noguchi, Yoko Ike, Nanami Yasui, Kazuki Furugori, Yuki Yamaguchi, Atsushi Toyoda, Yutaka Suzuki, Tatsuro Yamamoto, Noriko Saitoh, Shigeo Sato, Chieri Tomomori-Sato, Ronald C. Conaway, Joan W. Conaway, Hidehisa Takahashi

Figure S1 is related to Figure 1.
Figure S2 is related to Figure 1.
Figure S3 is related to Figure 1.
Figure S4 is related to Figure 1.
Figure S5 is related to Figure 2.
Figure S6 is related to Figure 4.
Figure S7 is related to Figure 5.
Figure S8 is related to Figure 6.
Figure S9 is related to Figure 6.
Figure S10 is related to Figure 7.
Figure S11 is related to Figure 7.
Figure S12 is related to Figure 7.
Figure S13 is related to Figure 8.
Figure S14 is related to Figure 9.

Oligonucleotide information of primers used for qPCR analysis, CRISPR vector construction and 4C-seq analysis

Figure S1

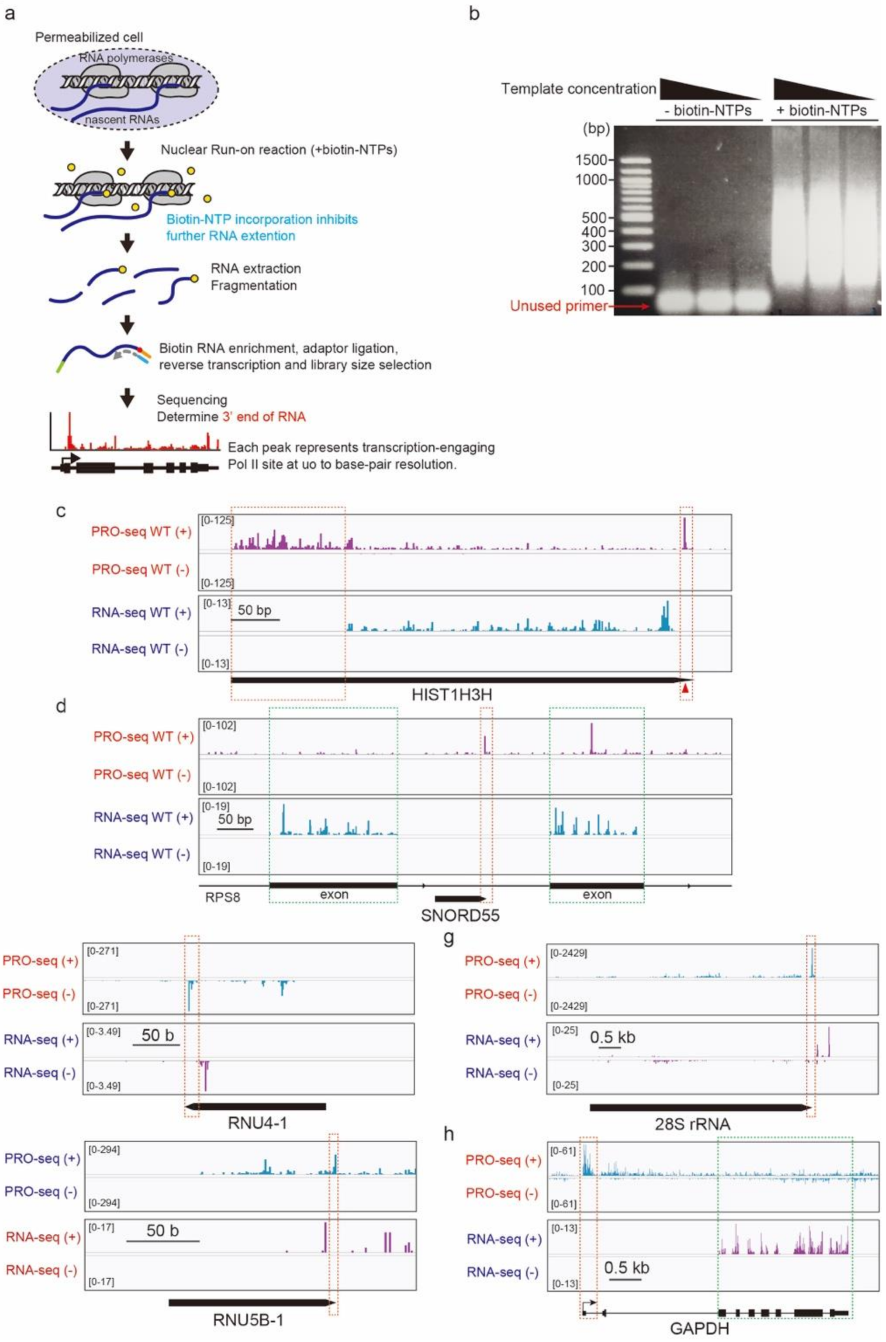

Supplementary Figure 1: Pol II pausing immediately upstream of transcript end sites of RDH genes was precisely detected by PRO-seq. (a) A schematic illustration of the PRO-seq flowchart. In PRO-seq, nuclear run-on reaction using biotin-NTPs is performed. Polymerases stop at the last incorporated biotin-NTP, which enables us to precisely detect 3′-end sites of nascent transcripts. (b) PRO-seq library preparation was performed with HEK293T cells using biotin-NTPs or without biotin-NTPs as a negative control. After a series of steps, PCR amplification of enriched cDNA was tested by agarose gel electrophoresis and SYBR Gold staining. (c–h) Genome browser tracks showing the distribution of 3′ ends of PRO-seq reads and those of total RNA-seq reads at *HIST1H3H* (c), *SNORD55* (d), *RNU4-1* (e), *RNU5B-1* (f), *28S rRNA* (g) and *GAPDH* (h) in wild-type HEK293T cells. The positive and negative strands of DNA are indicated by (+) and (–), respectively.

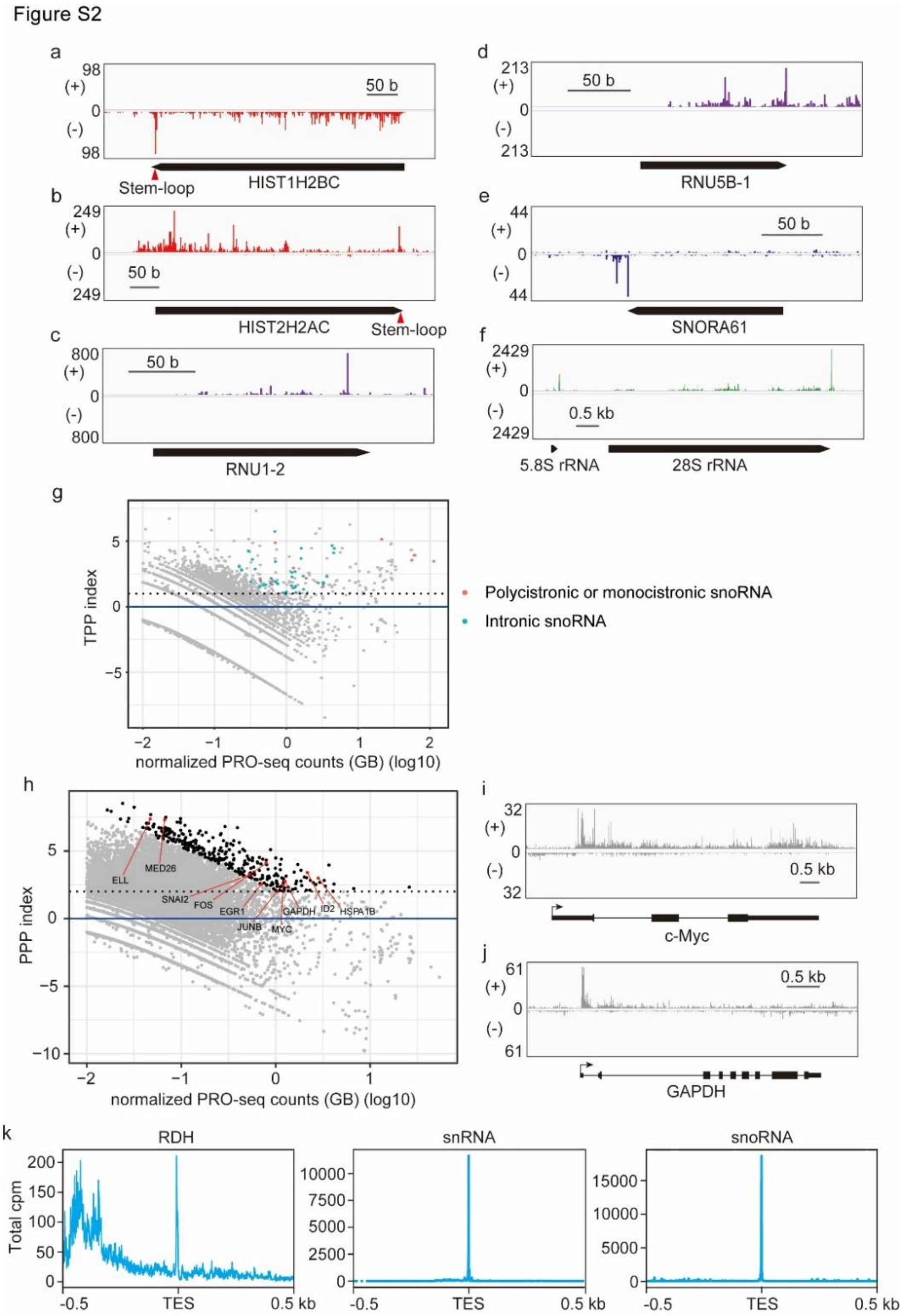

Supplementary Figure 2: Pol II paused at the TES proximal region of non-polyadenylated genes. (a–f) Genome browser tracks showing the distribution of PRO-seq reads at *HIST1H2BC* (a), *HIST2H2AC* (b), *RNU1-2* (c), *RNU5B-1* (d), *SNORA61* (e) and *28S rRNA* (f) in wild-type HEK293T cells. The positive and negative strands of DNA are indicated by (+) and (–), respectively. (g) Scatter plot showing TES proximal pausing (TPP) index relative to the normalized PRO-seq counts. Orange circles indicate poly- or mono-cistronic snoRNAs and green circles indicate intronic snoRNAs. (h) Scatter plot showing promoter proximal pausing (PPP) index relative to the normalized PRO-seq counts. Black circles indicate the genes showing a higher PPP index. (i, j) Genome browser tracks showing the distribution of PRO-seq reads at *c-Myc* and *GAPDH* in wild-type HEK293T cells. The positive and negative strands of DNA are indicated by (+) and (–), respectively. (k) Meta-gene analysis of PRO-seq reads around TESs of RDH genes, snRNA genes and snoRNA genes from RefGene data.

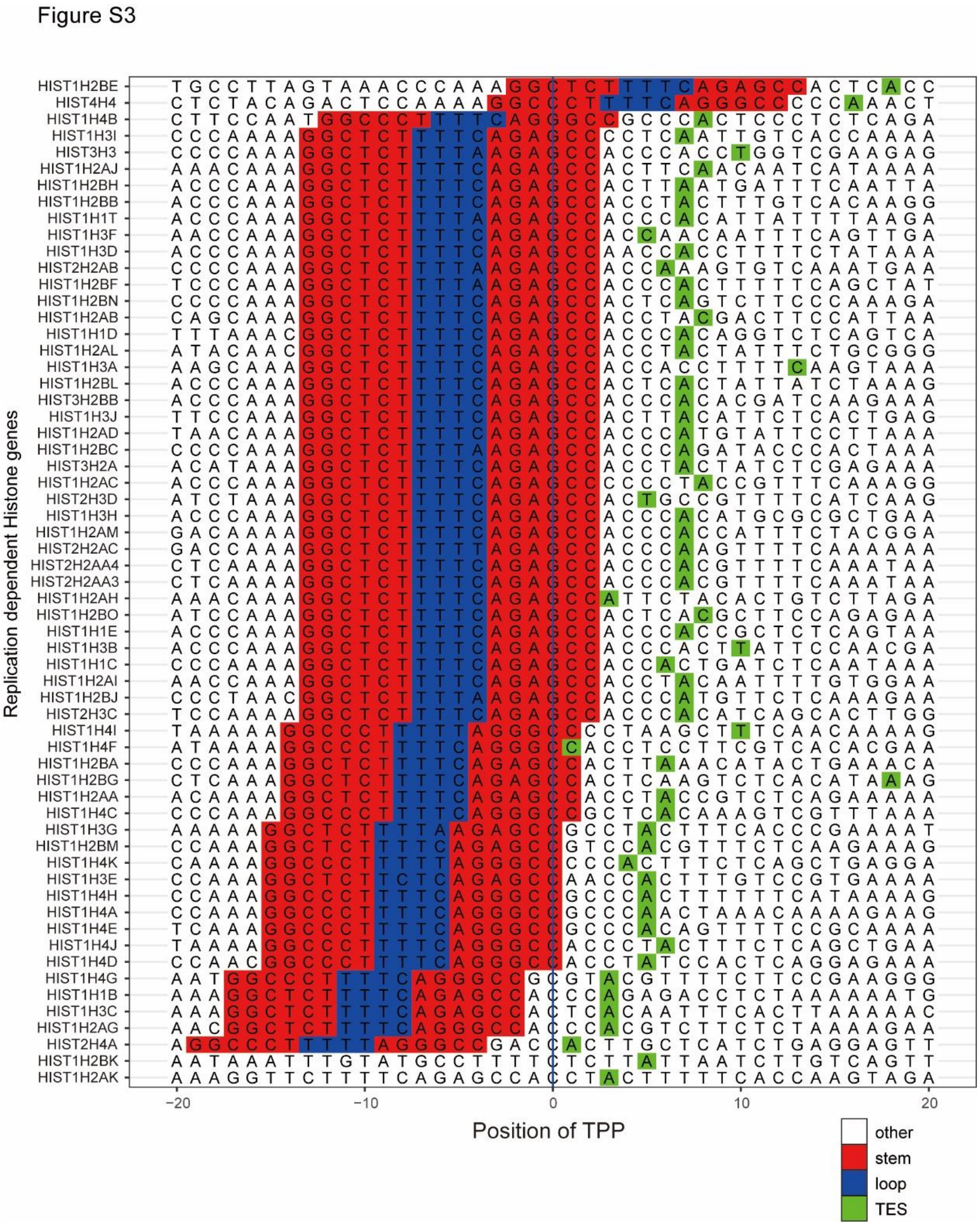

Supplementary Figure 3: Position of TPP at replication-dependent histone genes. Stem loop region (stem: red, loop: blue), TES (green) and the position of TPP (blue line: position 0) are indicated.

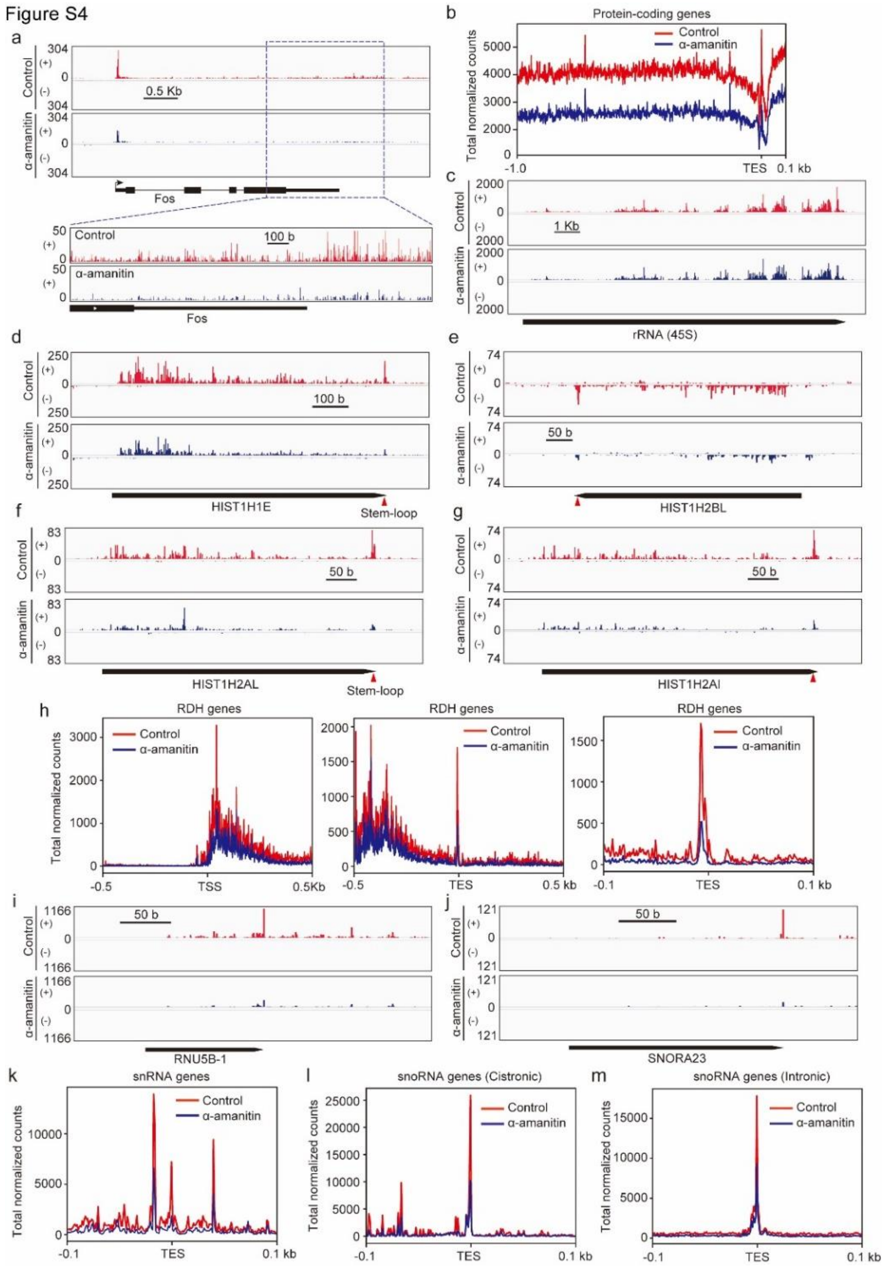

Supplementary Figure 4: PRO-seq in HEK293T cells in the presence of α-amanitin. HEK293T cells were treated with 10 µg/ml of α-amanitin for 2 h and cells were harvested for PRO-seq. Before the nuclear run-on reaction in PRO-seq, permeabilized cells were incubated with 10 µg/ml α-amanitin for 5 min, followed by nuclear run-on by the addition of biotin-NTPs as described in the Methods. (a) Genome browser tracks showing the distribution of PRO-seq reads at the *Fos* gene in control and α-amanitin-treated cells. The lower panel shows detailed genome browser tracks around the 3′ region of the *Fos* gene. The positive and negative strands of DNA are indicated by (+) and (–), respectively. (b) Meta-gene analysis of PRO-seq reads around the 3′ region of protein-coding genes. (c–g) Genome browser tracks showing the distribution of PRO-seq reads at rRNA (c), *HIST1H1E* (d), *HIST1H2BL* (e), *HIST1H2AL* (f), and *HIST1H2AI* (g) genes in control and α-amanitin-treated cells. The stem-loop sequence located immediately upstream of the TES of the RDH gene is indicated by a red arrowhead (d–g). (h) Meta-gene analysis of PRO-seq reads around the TSS and TES of RDH genes. (i, j) Genome browser tracks showing the distribution of PRO-seq reads at *RNU5B-1* (i) and *SNORA23* (j) genes in control and α-amanitin-treated cells. (k–m) Meta-gene analysis of PRO-seq reads around the TES of snRNA (k), cistronic snoRNA (l) and intronic snoRNA (m) genes.

Figure S5

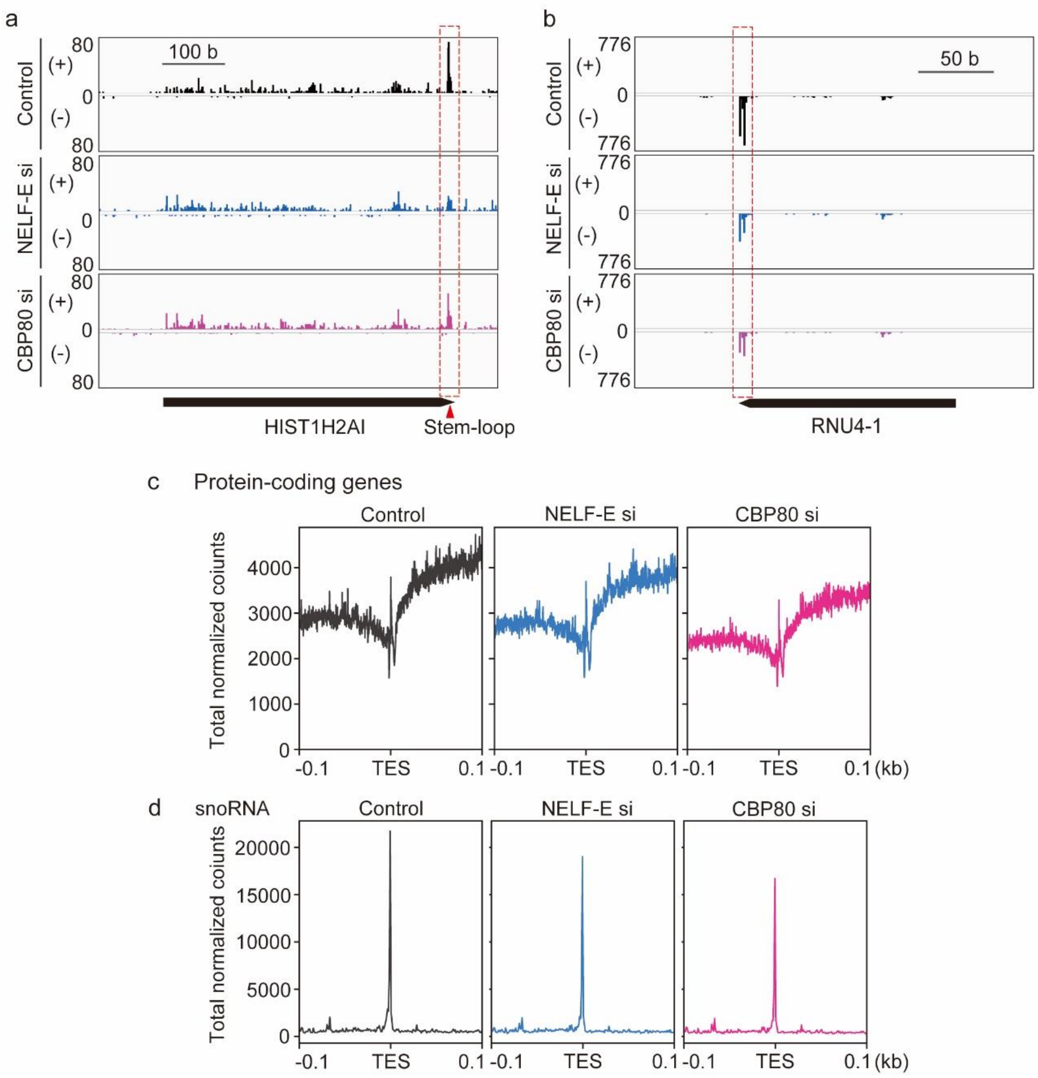

Supplementary Figure 5: Knockdown of NELF or CBP80 decreased the Pol II TPP at RDH genes. (a, b) Genome browser tracks showing the distribution of PRO-seq reads at *HIST1H2AI* (a) and *RNU4-1* (b) in control, NELF-knockdown (NELF-E si) and CBP80-knockdown (CBP80 si) cells. The positive and negative strands of DNA are indicated by (+) and (–), respectively. (c, d) Meta-gene analysis of PRO-seq reads around TESs of protein-coding genes (c) and snoRNA genes (d) in control, NELF-knockdown (NELF-E si) and CBP80-knockdown (CBP80 si) cells.

Figure S6

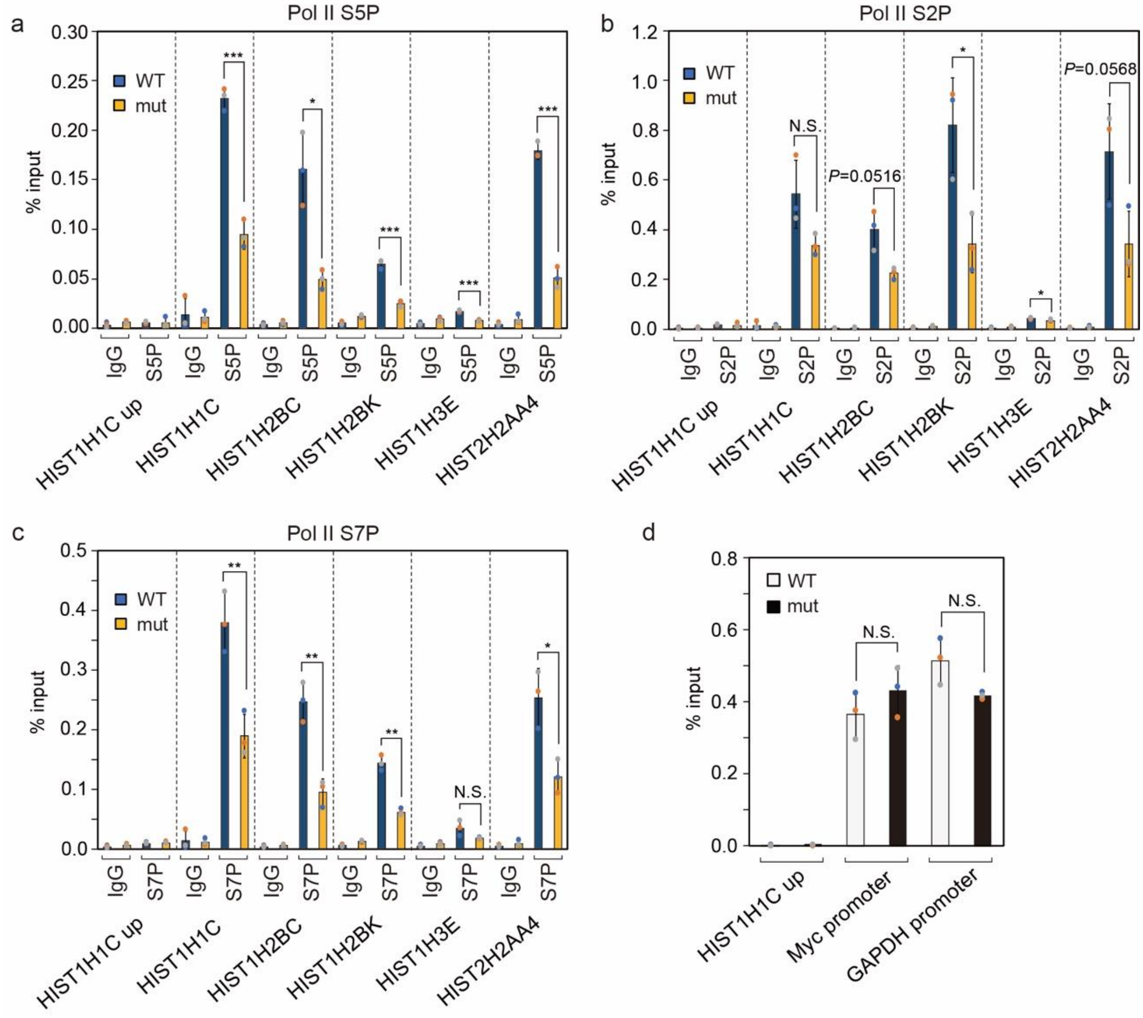

Supplementary Figure 6: Decreased occupancy of phosphorylated Pol II at RDH genes. (a–c) Occupancy of Ser5, Ser2 and Ser7 phosphorylated version of Pol II (ChIP/input) at each genomic locus was analysed in wild-type (WT) and EAF1-mutant (mut) cells. Ct values of each ChIP were normalized to that of input. The same IgG control is shown in (a), (b) and (c). Each value is the mean of three independent experiments. Error bars show standard deviation. The *P* values were determined by two-sided Student's *t* test (*, $P < 0.05$; **, $P < 0.01$; ***, $P < 0.001$). n = 3 biologically independent samples. N.S., not significant. Source data and exact *P* values are provided as a Source Data file. (d) Pol II occupancy (ChIP/input) at non-RDH genes in wild-type (WT) and EAF1-mutant (mut) cells. Each value is the mean of three independent experiments. Error bars show standard deviation. The *P* values were determined by two-sided Student's *t* test (*, $P < 0.05$; **, $P < 0.01$; ***, $P < 0.001$). n = 3 biologically independent samples. N.S., not significant. Source data and exact *P* values are provided as a Source Data file.

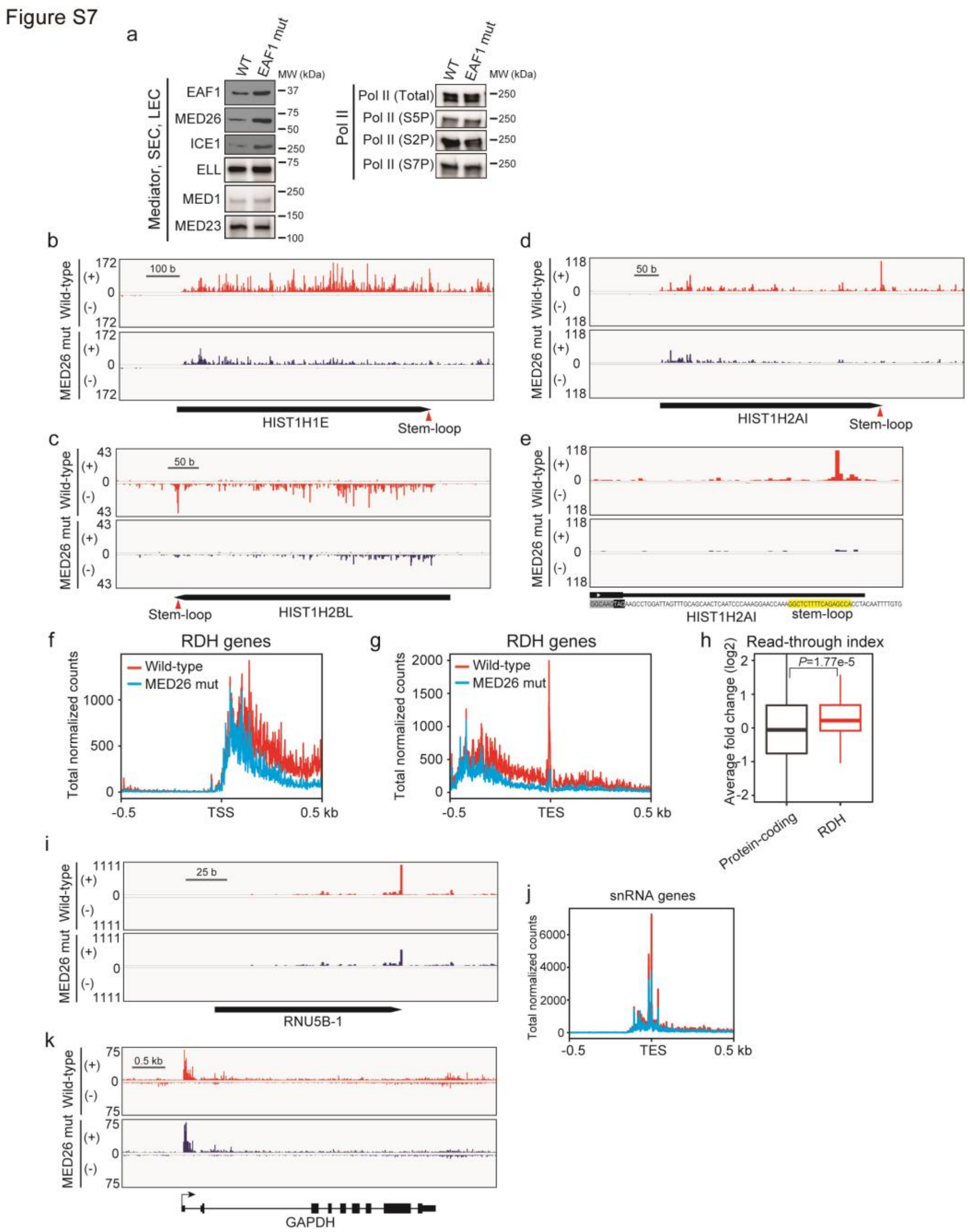

Supplementary Figure 7: TPP at RDH genes was diminished in mutant cell lines. (a) Western blot showing the components of Mediator, SEC, LEC and Pol II in wild-type (WT) and EAF1-mutant (EAF1 mut) HEK293T cells. (b–d) Genome browser tracks showing the distribution of PRO-seq reads at *HIST1H1E* (b), *HIST1H2BL* (c), and *HIST1H2AI* (d) genes in WT and MED26-mutant (MED26 mut) HEK293T cells. The stem-loop sequence located immediately upstream of TES of the RDH gene is indicated by a red arrow. (e) Detailed genome browser tracks showing the distribution of PRO-seq reads at the transcript end site of the *HIST1H2AI* gene in WT and MED26 mut HEK293T cells. The stem-loop sequence is highlighted in yellow. (f, g) Meta-gene analysis of PRO-seq reads around the TSS and TES of RDH genes in WT and MED26 mut HEK293T cells. (h) Boxplots representing the PRO-seq read-through index of protein-coding genes (n=68750, black) and RDH genes (n=136, red). Read-through ratio was determined using transcripts around TES, which have more than 10-fold PRO-seq signals normalized by spike-in control. The centre line of each boxplot represents the median. Upper and lower fences of each boxplot represent upper and lower quartiles, respectively. The *P* values were determined by two-sided Wilcoxon's signed-rank test. Source data are provided as a Source Data file. (i) Genome browser tracks showing the distribution of PRO-seq reads at *RNU5B-1* in WT and MED26 mut HEK293T cells. (j) Meta-gene analysis of PRO-seq reads around the TES of snRNA genes in WT and MED26 mut HEK293T cells. (k) Genome browser tracks showing the distribution of PRO-seq reads at *GAPDH* in WT and MED26 mut HEK293T cells.

Figure S8

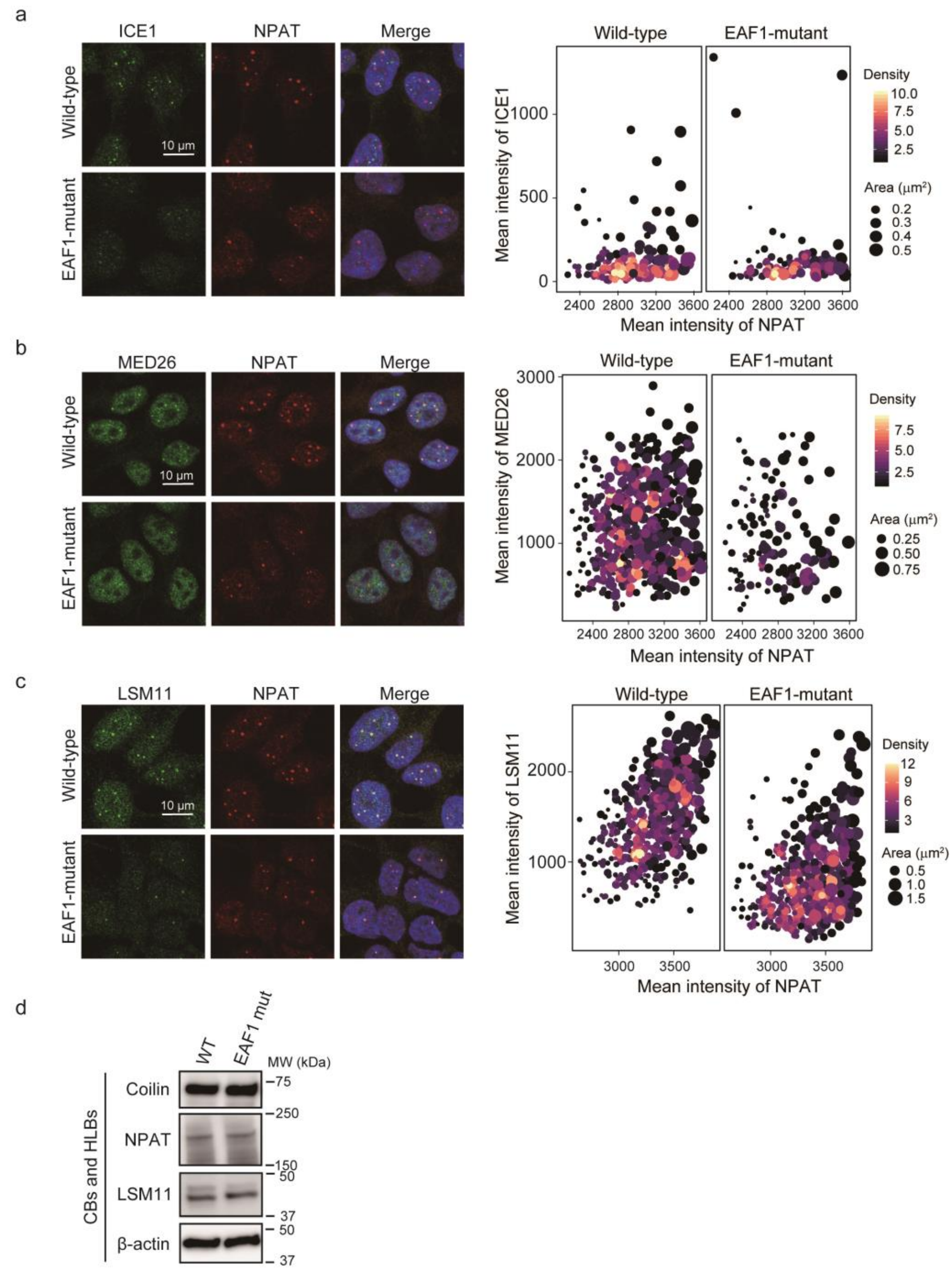

Supplementary Figure 8: Point mutation of EAF1 decreased associations of ICE1, MED26 and LSM11 with HLBs in cells. (a–c) Wild-type HEK293T cells and EAF1-mutant HEK293T cells were fixed with paraformaldehyde, and immunofluorescence staining for ICE1 (green) and NPAT (red) (a), MED26 (green) and NPAT (red) (b), and LSM11 (green) and NPAT (red) (c) was performed. Scale bar, 10 µm. Evaluation of HLB associations of ICE1, MED26 and LSM11 is shown in the right panels. The NPAT particles were extracted from images. The area of NPAT particles occupied by ICE1, MED26 and LSM11 particles and the intensity of the particles of NPAT, ICE1, MED26 and LSM11 were calculated. The density shows the degree to which dots overlap with others. (d) Protein expression levels in wild-type and EAF1-mutant HEK293T cells. Western blot showing the components of CBs and HLBs in wild-type (WT) and EAF1-mutant (EAF1 mut) HEK293T cells.

Figure S9

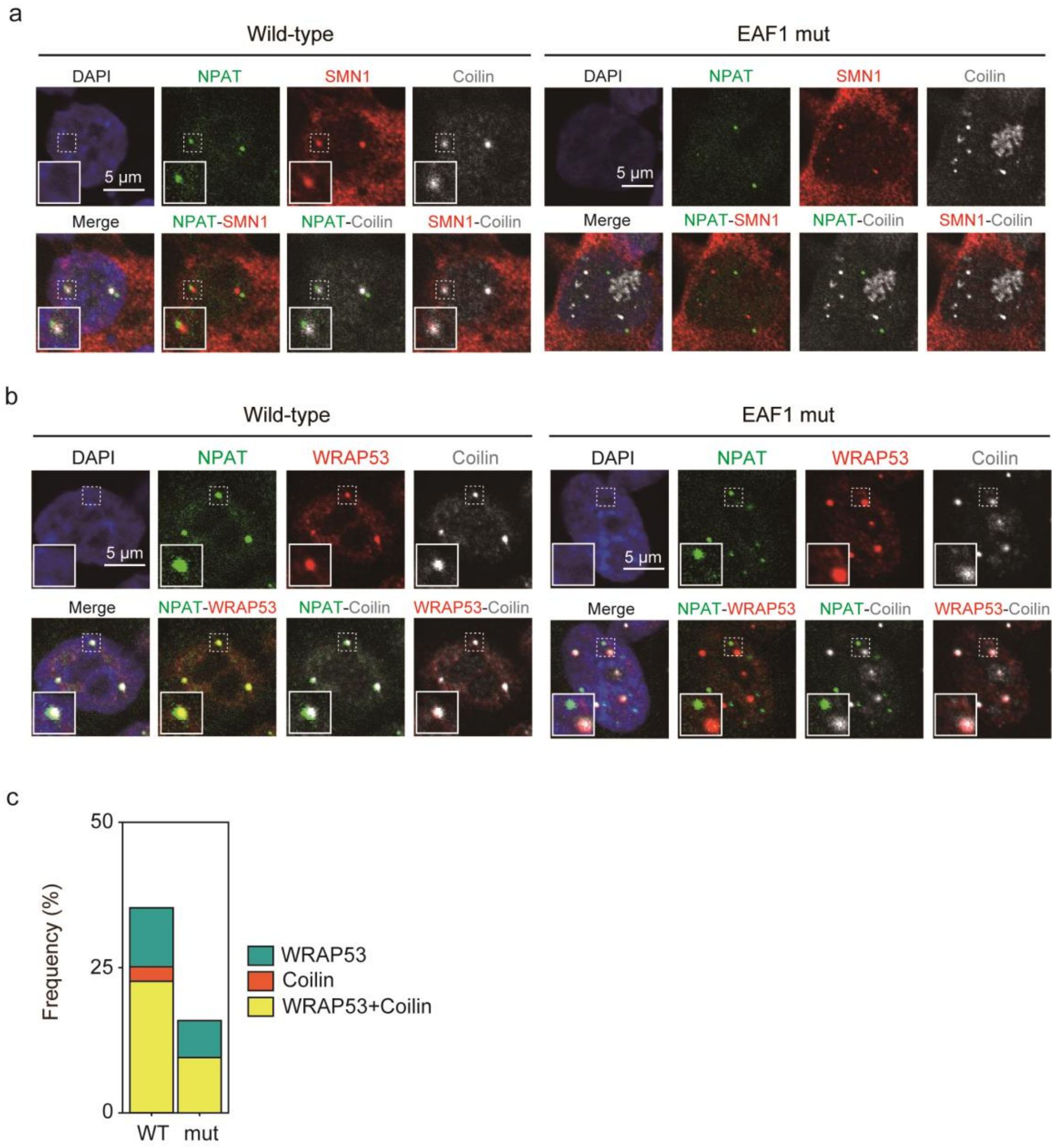

Supplementary Figure 9: Association of CBs with HLBs was decreased in EAF1-mutant cells. (a, b) Wild-type (WT) and EAF1-mutant (EAF1 mut) HEK293T cells were fixed with paraformaldehyde and subjected to triple immunofluorescence staining. Co-staining of SMN1 (a, red) and WRAP53 (b, red) with NPAT (green) and Coilin (grey) was performed. Scale bar, 5 μm. (c) The frequency of NPAT colocalization with WRAP53 and/or Coilin in WT and EAF1-mutant (mut) HEK293T cells is shown. The frequency of colocalization was calculated using n=481 WT nuclei (n=412 NPAT particles) and n=231 mut nuclei (n=126 NPAT particles). Source data are provided as a Source Data file.

Figure S10

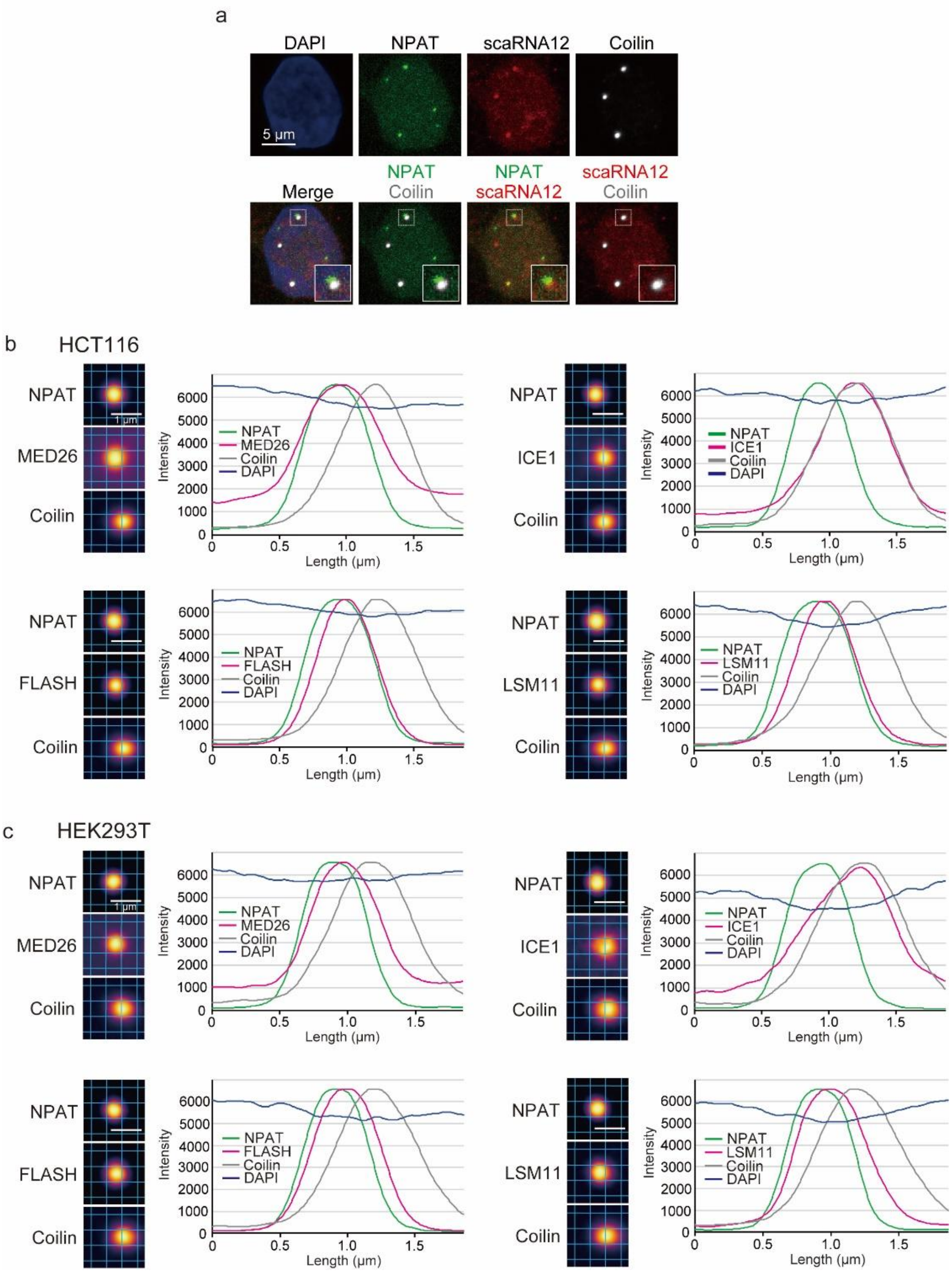

Supplementary Figure 10: Relative localization patterns of Mediator, LEC and 3′-end processing factors to HLB-CB. (a) Detection of the association of scaRNA-containing CBs with HLBs. HeLa cells were fixed with paraformaldehyde containing 0.5% Triton X-100 and subjected to RNA-FISH. Four-colour microscopy images are shown. NPAT (green) and Coilin (gray) were stained using specific antibodies, and scaRNA12 (red) was stained using Cy3-labelled probe. Enlarged images for representative particles are shown in the insets in each image. Scale bar, 5 µm. (b, c) Wild-type HCT116 cells (b) and HEK293T cells (c) were fixed with paraformaldehyde, and immunofluorescence staining for MED26, ICE1, FLASH and LSM11 together with NPAT and Coilin was performed. Averaged signals of immunofluorescence centred at the NPAT signal and respective line plots are shown for each immunofluorescence experiment. Scale bar, 1 µm. Source data are provided as a Source Data file.

Figure S11

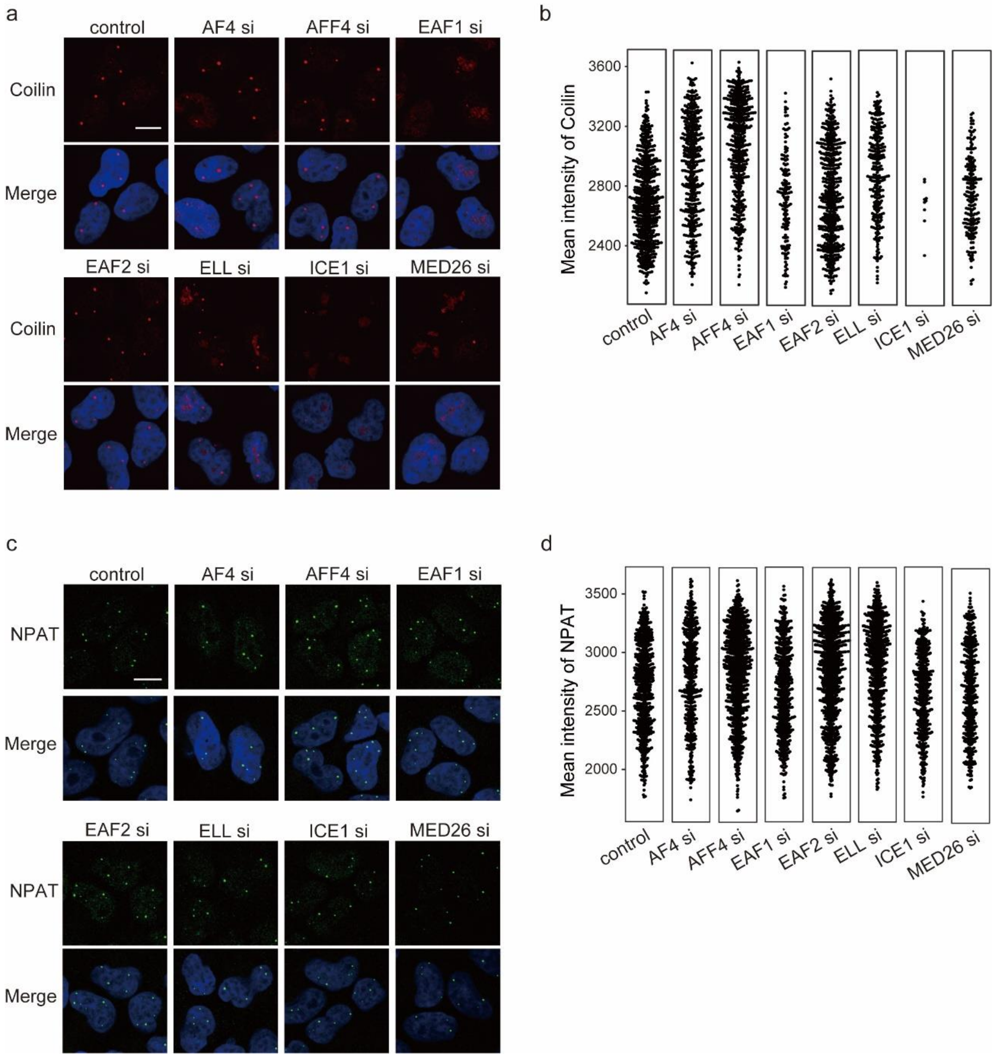

Supplementary Figure 11: Evaluation of the formation of CBs and HLBs upon knockdown of the components of SEC or LEC. (a) Effect on CB formation of the knockdown of SEC or LEC components. HeLa cells were transfected with the indicated siRNAs. At 48 h after siRNA transfection, the cells were fixed with paraformaldehyde and subjected to immunofluorescence staining using anti-Coilin antibodies. Scale bar, 10 µm. (b) Quantification of Coilin signal intensity in siRNA-transfected HeLa cells. The mean signal intensity of Coilin for each particle was determined, and beeswarm plots for each condition are shown. Coilin signals were evaluated from around 300 nuclei for each condition (control, n=308; AF4 si, n=279; AFF4 si, n=306; EAF1 si, 316; EAF2 si, n=295; ELL si, n=271; ICE1 si, n=283; MED26 si, n=295). (c) HeLa cells were transfected with the indicated siRNAs. At 48 h after siRNA transfection, the cells were fixed with paraformaldehyde and subjected to immunofluorescence staining using anti-NPAT antibodies. Scale bar, 10 µm. (d) Quantification of signal intensity of NPAT in siRNA-transfected HeLa cells. Mean signal intensity of NPAT for each particle was determined, and beeswarm plots for each condition are shown. NPAT signals were evaluated from around 300 nuclei for each condition (control, n=308; AF4 si, n=279; AFF4 si, n=306; EAF1 si, 316; EAF2 si, n=295; ELL si, n=271; ICE1 si, n=283; MED26 si, n=295).

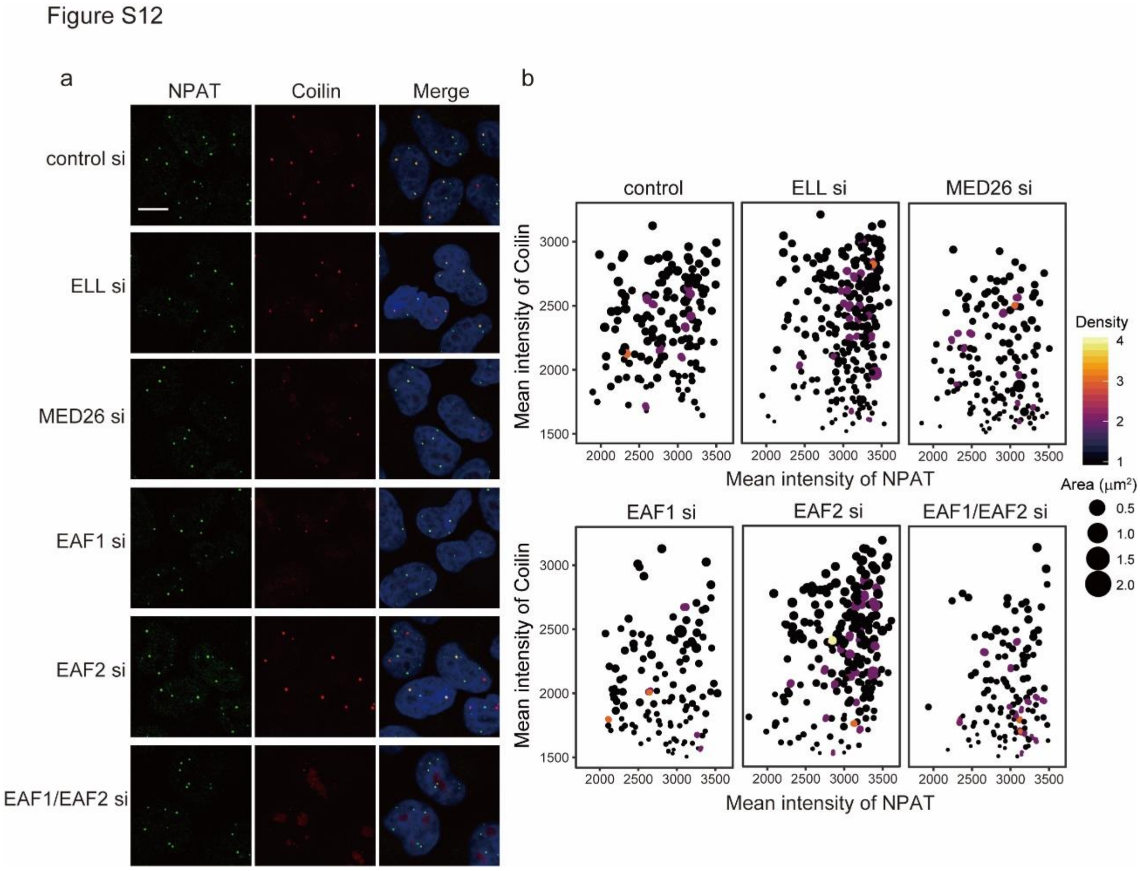

Supplementary Figure 12. MED26 and EAF1 were required for CBs' association with HLBs. (a) Knockdown of MED26 and EAF1 affects CBs' association with HLBs. HeLa cells were transfected with the indicated siRNAs. At 48 h after siRNA transfection, the cells were fixed with paraformaldehyde and subjected to immunofluorescence staining using anti-Coilin and anti-NPAT antibodies. Scale bar, 10 μm. (b) Evaluation of CB–HLB association in each cell transfected with the indicated siRNAs. NPAT-associating Coilin particles were extracted from images, and mean intensities of the particles of NPAT and Coilin were plotted. Signal intensities were evaluated from around 300 nuclei for each condition (control, n=308; ELL si, n= 271; MED26 si, n=295; EAF1, n=316; EAF2 si, n=295; EAF1/EAF2 si, n=271).

Figure S13

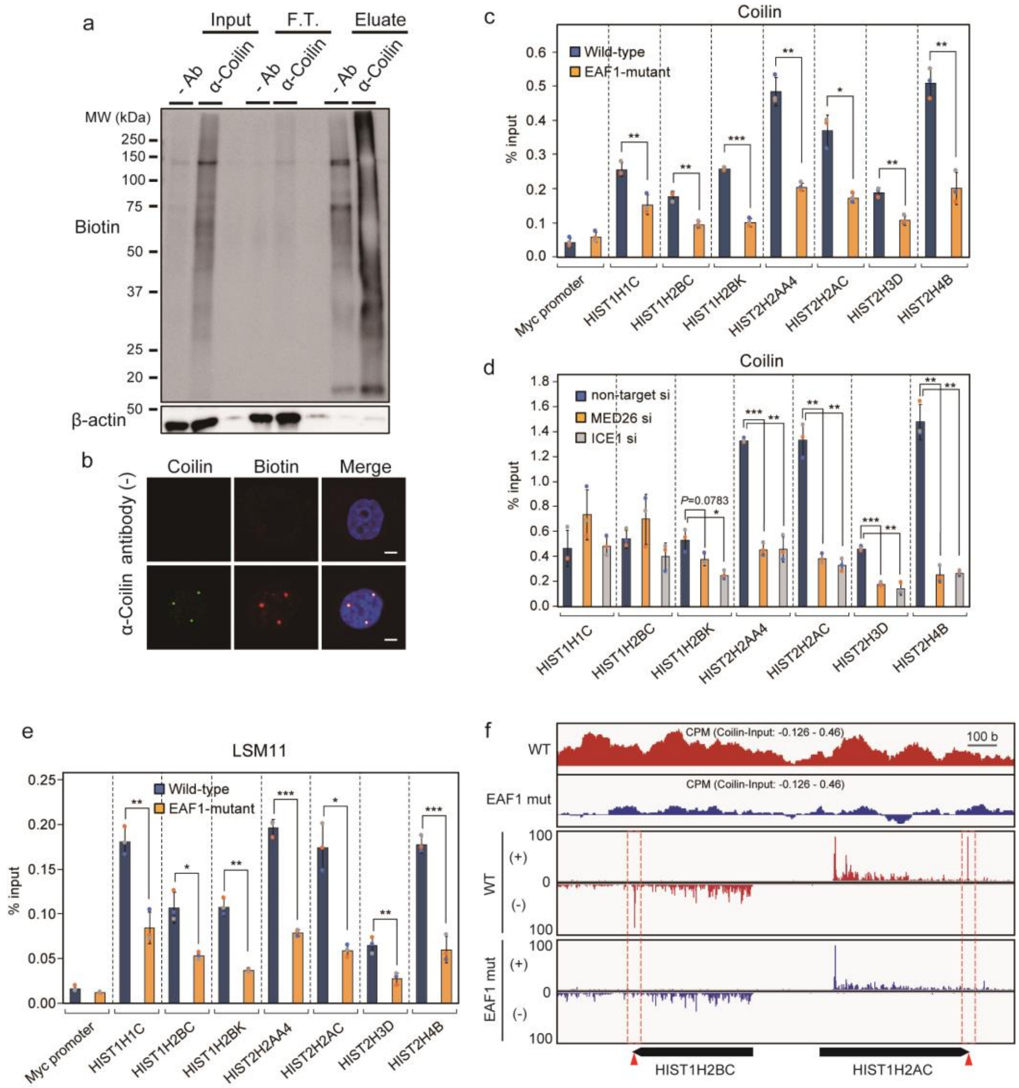

Supplementary Figure 13: *In situ* biotinylation of CB-proximal region using anti-Coilin antibodies. (a) Western blot showing anti-Coilin antibody-specific biotinylation. *In situ* biotinylation using anti-Coilin antibodies was performed with HeLa cells, and avidin-purified proteins (Eluate) and flow-through fraction (F.T.) were subjected to western blotting. (b) Fluorescence detection of biotinylated proteins. CB-proximal regions were specifically biotinylated by an *in situ* biotinylation method. After *in situ* biotinylation using anti-Coilin antibodies, biotinylated proteins were detected by Alexa-594-conjugated streptavidin. Scale bar, 10 μm. (c, d) *In situ* biotinylation using anti-Coilin antibodies was performed with wild-type HEK293T and EAF1-mutant HEK293T cells (c) or HeLa cells transfected with the indicated siRNAs (d). The biotinylated chromatins were purified, and the associated DNAs were analysed by qPCR. Each value is the mean of three independent experiments. Error bars show standard deviation. The *P* values were determined by two-sided Student's *t* test (*, $P < 0.05$; **, $P < 0.01$; ***, $P < 0.001$). n = 3 biologically independent samples. Source data and exact *P* values are provided as a Source Data file. (e) U7 snRNP (LSM11)-associated chromatin was purified using *in situ* biotinylation with anti-LSM11 antibodies followed by avidin precipitation, and qPCR was performed. Each value is the mean of three independent experiments. Error bars show standard deviation. The *P* values were determined by two-sided Student's *t* test (*, $P < 0.05$; **, $P < 0.01$; ***, $P < 0.001$). n = 3 biologically independent samples. Source data and exact *P* values are provided as a Source Data file. (f) Decreased association of CBs with RDH genes was accompanied by decreased TPP at RDH genes. Genome browser tracks showing the distribution of *in situ* biotinylation-seq reads and PRO-seq peaks around *HIST1H2BC* and *HIST1H2AC* loci.

Figure S14

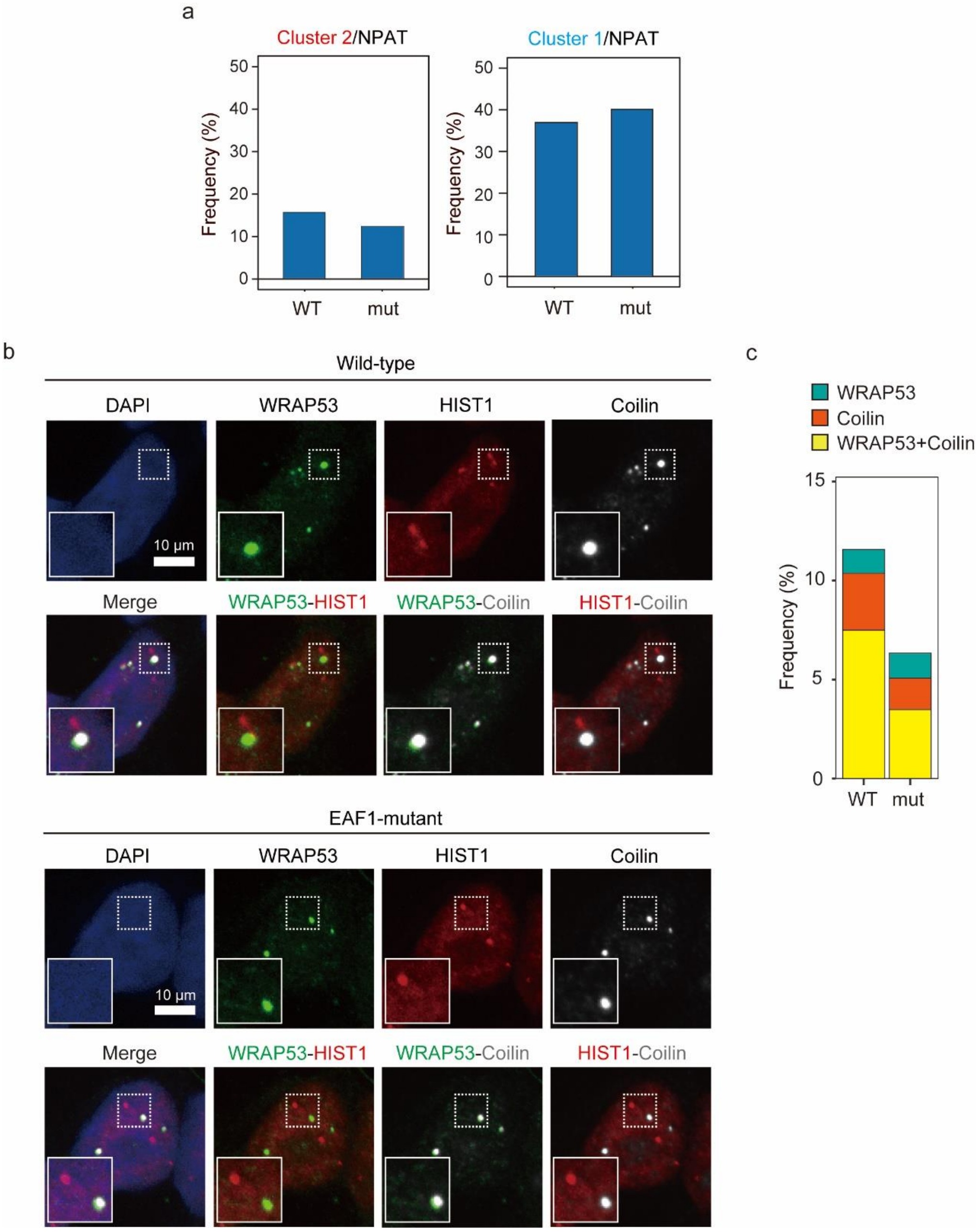

Supplementary Figure 14: CB association with RDH gene clusters was affected in EAF1-mutant cells, but HLB association with RDH gene clusters was not. (a) Four-colour DNA-fluorescence *in situ* hybridization was performed with wild-type (WT) and EAF1-mutant (mut) cells, and the frequencies of HLB (NPAT) association with each RDH gene cluster in WT and mut cells were determined. The frequencies were calculated from n=752 NPAT particles in WT cells and n=516 NPAT particles in mut cells. Source data are provided as a Source Data file. (b, c) RDH gene cluster 1 (HIST1), WRAP53 and Coilin in WT and mut cells were detected by four-colour DNA fluorescence *in situ* hybridization. Scale bar, 5 μm (b). The frequencies of RDH gene cluster 1 association with WRAP53 and Coilin in WT and mut cells were determined (c). The frequencies were calculated from n=103 WT nuclei (n=370 RDH gene foci) and n=53 mut nuclei (n=484 RDH gene foci). Source data are provided as a Source Data file.

Oligonucleotide information of primers used for qPCR analysis, CRISPR vector construction and 4C-seq analysis

Primers used for qPCR analysis

Human HIST1H1C upstream

5′-ATTCAGAGCTCTTAGGCGAAAT-3′ (forward)

5′-CATTGACACATACACAAGCTCAC-3′ (reverse)

Human HIST1H1C

5′-CGGCCACTGTAACCAAGAA-3′ (forward)

5′-ACAGCCTTAGCAGCACTTT-3′ (reverse)

Human HIST1H2BC

5′-GACACTGGCATCTCTTCCAA-3′ (forward)

5′-GTCGAGCGCTTGTTGTAATG-3′ (reverse)

Human HIST1H2BK

5′-CTCTAAGGCCATGGGAATCAT-3′ (forward)

5′-GTCGAGCGCTTGTTGTAATG-3′ (reverse)

Human HIST1H3E

5′-GCGAGAAATAGCTCAGGACTT-3′ (forward)

5′-CACGCGTTTGGCATGAATAG-3′ (reverse)

Human HIST1H3B

5′-TCCGAATCAGCAACTCGGTC-3′ (forward)

5′-AGACAGCTCGGAAATCCACC-3′ (reverse)

Human HIST2H2AA4

5′-GCTCGGGACAACAAGAAGA-3′ (forward)

5′-CAGTACGGCCTGGATGTTAG-3′ (reverse)

Human HIST2H4B

5′-CGGCGTTAAGCGGATCTCT-3′ (forward)

5′-GTGACTGCGTCCCGAATCA-3′ (reverse)

Human HIST2H2AC

5′-GCGCTATTTCGTGATCAGCC-3′ (forward)

5′-CCTGTTGCCCAAGAAAACGG-3′ (reverse)

Human HIST2H3D

5′-TCGAAGACACGAACCTGTGC-3′ (forward)

5′-CGGTACAGCTGCTCTTGATGA-3′ (reverse)

Human Myc promoter region

5′-TTCTCAGAGGCTTGGCGGGAAA-3′ (forward)

5′-CTGCCTCTCGCTGGAATTACTACA-3′ (reverse)

Human GAPDH promoter region

5′-CTCAAGACCTTGGGCTGGG-3′ (forward)

5′-TCGAACAGGAGGAGCAGAGA-3′ (reverse)

Human TRIM38 downstream region

5′-AGTGCTGTCCTTACATCGCA-3′ (forward)

5′-ATCCCACTAGCCCCTCATCC-3′ (reverse)

Oligonucleotides used for CRISPR vector construction

EAF1 sgRNA-Sense

CACCGACTTGCAGTTGAGTGAGTC

EAF1 sgRNA-Antisense

AAACGACTCACTCAACTGCAAGTC

CRISPR knock-in template oligo

catgtttgtgcaccaaaaagtaggtttcgaaagatccagcactagtcatcactTGCTGCTGCTGCctcactcaactgcaagtcattt

cctatttaaaaaaaaaaataaagtaaaatcata

Oligonucleotides used for generation of 4C-seq templates

4C-reading primer for HIST1H2BK

TACACGACGCTCTTCCGATCTACTTTCACTCTTCACCTTATTTGCATG

4C-non-reading primer for HIST1H2BK

ACTGGAGTTCAGACGTGTGCTCTTCCGATCTGAGTACCTGACCGCTGAGATC

4C-reading primer for HIST2H2AB

TACACGACGCTCTTCCGATCTCCACATTAAAGCCCCTTATTTGCATG

4C-non-reading primer for HIST2H2AB

ACTGGAGTTCAGACGTGTGCTCTTCCGATCTCAAAGTGTCAAATGAAGGGCTGATC
