# Supplementary material for: The 3′ Pol II pausing at replication-dependent histone genes is regulated by Mediator through Cajal bodies’ association with histone locus bodies
Source: Nat Commun. 2022 May 25;13:2905. doi: 10.1038/s41467-022-30632-w (PMC9133132; doi:10.1038/s41467-022-30632-w)
Supplement: Supplementary file 3 — Description of additional Supplementary File [file 41467_2022_30632_MOESM3_ESM.pdf]

Descriptions of additional supplementary data files

Supplementary Data 1 Supplementary Data 1 is related to Figure 1. The data include TPP genes identified in MED26 ChIP-seq analysis. Peak calling of MED26 ChIPseq was performed using MACS with the default settings. The MED26 ChIPseq data are deposited in GEO under accession number GSE121024.

Supplementary Data 2 Supplementary Data 2 is related to Figure 4. RNA-seq of ribo-depleted libraries from HEK293T cells and EAF1-mutant cell lines. The data include transcripts exhibiting decreased abundance ($\log 2 \leq 1$, adjusted P-value ≤ 0.01) in ribodepleted libraries. The P values were determined by two-sided Wilcoxon’s signed-rank test.
