# Supplementary material for: The 3′ Pol II pausing at replication-dependent histone genes is regulated by Mediator through Cajal bodies’ association with histone locus bodies
Source: Nat Commun. 2022 May 25;13:2905. doi: 10.1038/s41467-022-30632-w (PMC9133132; doi:10.1038/s41467-022-30632-w)
Supplement: Supplementary file 6 — Reporting Summary [file 41467_2022_30632_MOESM6_ESM.pdf]

nature portfolio

Corresponding author(s): Hidehisa Takahashi

Last updated by author(s): Apr 26, 2022

Reporting Summary

Nature Portfolio wishes to improve the reproducibility of the work that we publish. This form provides structure for consistency and transparency in reporting. For further information on Nature Portfolio policies, see our Editorial Policies and the Editorial Policy Checklist.

Statistics

For all statistical analyses, confirm that the following items are present in the figure legend, table legend, main text, or Methods section.

n/a	Confirmed	
☐	☒	The exact sample size (*n*) for each experimental group/condition, given as a discrete number and unit of measurement
☐	☒	A statement on whether measurements were taken from distinct samples or whether the same sample was measured repeatedly
☐	☒	The statistical test(s) used AND whether they are one- or two-sided<br>*Only common tests should be described solely by name; describe more complex techniques in the Methods section.*
☐	☒	A description of all covariates tested
☐	☒	A description of any assumptions or corrections, such as tests of normality and adjustment for multiple comparisons
☐	☒	A full description of the statistical parameters including central tendency (e.g. means) or other basic estimates (e.g. regression coefficient) AND variation (e.g. standard deviation) or associated estimates of uncertainty (e.g. confidence intervals)
☐	☒	For null hypothesis testing, the test statistic (e.g. *F*, *t*, *r*) with confidence intervals, effect sizes, degrees of freedom and *P* value noted<br>*Give P values as exact values whenever suitable.*
☒	☐	For Bayesian analysis, information on the choice of priors and Markov chain Monte Carlo settings
☐	☒	For hierarchical and complex designs, identification of the appropriate level for tests and full reporting of outcomes
☒	☐	Estimates of effect sizes (e.g. Cohen's *d*, Pearson's *r*), indicating how they were calculated

Our web collection on statistics for biologists contains articles on many of the points above.

Software and code

Policy information about availability of computer code

Data collection	Huygens software (Scientific Volume Imaging B.V.) was used for deconvolution of super-resolution immunofluorescence images.
Data analysis	For RNA-seq, raw reads from sequencing were demultiplexed allowing up to one mismatch using Illumina bcl2fastq2 v2.18. Adapter sequences were trimmed using Trim Galore (v 0.64_dev), and paired-end reads were mapped to human genome GRCH38 with hisat2 (version 2.3.4.1), using Ensembl gene annotation GRCh38.p13. Read counts were obtained using Rsubread (version 2.2.6). Differential gene expression analysis was performed using R (v. 4.0.2) package DESeq2 (v. 1.28.1). Genes with an adjusted P value <0.01 and absolute log2 fold change >0.5 were included in the downstream analysis. A read-through plot was created with modified ngsplot (2.6.1) to show a logarithmic y-axis. HIST2H2AC, HIST2H2AB and HIST1H3J were excluded from this analysis because their transcript end sites (TESs) were too near to each other or the TES positions appeared to be incorrect.<br><br>For PRO-seq, raw data of sequences were trimmed using Trim Galore (v 0.64_dev), and the reads were mapped to the human genome GRCh38 and Drosophila melanogaster genome build5.41 using the Bowtie2 alignment tool (version 2.3.5.1) with the default settings. Read counts were normalized according to the genomic coverage of mapped Drosophila reads using bedtools (version 2.29.2) and samtools (version 1.7). Pileup tracks of the last base pair of the reads were generated using bedtools (version 2.29.2) and used for downstream analysis. For read-through analysis, read counts from TSS to +200 bp downstream of it and from TES to +200 bp downstream of it were piled up by R package rtracklayer (version 1.48.0). refGene of GRCh38.p13 was used as a reference of transcripts. Then, the read-through ratio was defined using the following equation: (from TES to TES+200 bp) / (from TSS to TSS+200 bp). Meta-gene plots were generated with deeptools (version 3.3.1) using refGene as a reference. Multiple transcripts sharing the same TSS or TES were analysed as a single gene transcript.<br>For PRO-seq data analysis of α-amanitin-treated samples, the read counts were normalized according to million rRNA reads to evaluate effect of α-amanitin on Pol II-dependent PRO-seq signals. rRNA reads were defined as reads mapped to chrUn_GL000220v1: 105424 -118780 and counted using bedtools.<br><br>For ChIP-seq and in situ biotinylation-seq, adapter sequences were trimmed using Trim Galore (v 0.64_dev), and reads were mapped to

human genome GRCH38 and Drosophila melanogaster genome build5.41 (for spike-in reads of in situ biotinylation-seq) using the Bowtie2 alignment tool (version 2.3.5.1) with the default settings. The mapped reads from each ChIP-seq and in situ biotinylation-seq dataset were subjected to count per million (CPM) normalization and then used for downstream analysis. IP/input or IP-input enrichment per gene was calculated with R (v. 4.0.2) and its package rtracklayer (version 1.48.0) using the Ensembl GRCH38.92 gene annotation model.

For 4C-seq, pipe4C was used with the default setting for the data analysis. R1 files of paired-end read files were used for 4C-seq data analysis.

For immunofluorescence image analysis, quantification of the signal intensity of the particles was performed using ImageJ Fiji software.

For manuscripts utilizing custom algorithms or software that are central to the research but not yet described in published literature, software must be made available to editors and reviewers. We strongly encourage code deposition in a community repository (e.g. GitHub). See the Nature Portfolio guidelines for submitting code & software for further information.

Data

Policy information about availability of data

All manuscripts must include a data availability statement. This statement should provide the following information, where applicable:

- Accession codes, unique identifiers, or web links for publicly available datasets
- A description of any restrictions on data availability
- For clinical datasets or third party data, please ensure that the statement adheres to our policy

GEO accession code: GSE164144
Please go to https://www.ncbi.nlm.nih.gov/geo/query/acc.cgi?acc=GSE164144 and enter token "ixkxomaofncdxkl" into the box.

A list of Figures that have associated raw data
RNAseq_293T-WT_Rep1_R1.fastq.gz: Figure 4a, b, e, f, Figure S1c-h
RNAseq_293T-WT_Rep1_R2.fastq.gz: Figure 4a, b, e, f, Figure S1c-h
RNAseq_293T-WT_Rep2_R1.fastq.gz: Figure 4a, b, e, f, Figure S1c-h
RNAseq_293T-WT_Rep2_R2.fastq.gz: Figure 4a, b, e, f, Figure S1c-h
RNAseq_293T-WT_Rep3_R1.fastq.gz: Figure 4a, b, e, f, Figure S1c-h
RNAseq_293T-WT_Rep3_R2.fastq.gz: Figure 4a, b, e, f, Figure S1c-h
RNAseq_293T-EAF1mut_Rep1_R1.fastq.gz: Figure 4a, b, e, f
RNAseq_293T-EAF1mut_Rep1_R2.fastq.gz: Figure 4a, b, e, f
RNAseq_293T-EAF1mut_Rep2_R1.fastq.gz: Figure 4a, b, e, f
RNAseq_293T-EAF1mut_Rep2_R2.fastq.gz: Figure 4a, b, e, f
RNAseq_293T-EAF1mut_Rep3_R1.fastq.gz: Figure 4a, b, e, f
RNAseq_293T-EAF1mut_Rep3_R2.fastq.gz: Figure 4a, b, e, f
PROseq_293T-WT_Rep1_R1.fastq.gz: Figure 1a-h, Figure 5a-k, Figure 8j, Figure S1c-h, Figure S2a-k, Figure S3, Figure S13f
PROseq_293T-WT_Rep1_R2.fastq.gz: Figure 1a-h, Figure 5a-k, Figure 8j, Figure S1c-h, Figure S2a-k, Figure S3, Figure S13f
PROseq_293T-WT_Rep2_R1.fastq.gz: Figure 1a-h, Figure 5a-k, Figure 8j, Figure S1c-h, Figure S2a-k, Figure S3, Figure S13f
PROseq_293T-WT_Rep2_R2.fastq.gz: Figure 1a-h, Figure 5a-k, Figure 8j, Figure S1c-h, Figure S2a-k, Figure S3, Figure S13f
PROseq_293T-EAF1mut_Rep1_R1.fastq.gz: Figure 5a-k, Figure 8j, Figure S13f
PROseq_293T-EAF1mut_Rep1_R2.fastq.gz: Figure 5a-k, Figure 8j, Figure S13f
PROseq_293T-EAF1mut_Rep2_R1.fastq.gz: Figure 5a-k, Figure 8j, Figure S13f
PROseq_293T-EAF1mut_Rep2_R2.fastq.gz: Figure 5a-k, Figure 8j, Figure S13f
ChIPseq_293T-WT_ELL_Input_R1.fastq.gz: Figure 4b
ChIPseq_293T-WT_ELL_Input_R2.fastq.gz: Figure 4b
ChIPseq_293T-WT_ELL_IP_Rep1_R1.fastq.gz: Figure 4b
ChIPseq_293T-WT_ELL_IP_Rep1_R2.fastq.gz: Figure 4b
ChIPseq_293T-WT_ELL_IP_Rep2_R1.fastq.gz: Figure 4b
ChIPseq_293T-WT_ELL_IP_Rep2_R2.fastq.gz: Figure 4b
4Cseq_293T-EAF1mut_HIST1H2BK-bait_Rep1_R1.fastq.gz: Figure 9c, d
4Cseq_293T-EAF1mut_HIST1H2BK-bait_Rep1_R2.fastq.gz: Figure 9c, d
4Cseq_293T-EAF1mut_HIST1H2BK-bait_Rep2_R1.fastq.gz: Figure 9c, d
4Cseq_293T-EAF1mut_HIST1H2BK-bait_Rep2_R2.fastq.gz: Figure 9c, d
4Cseq_293T-EAF1mut_HIST2H2AB-bait_Rep1_R1.fastq.gz: Figure 9e, f
4Cseq_293T-EAF1mut_HIST2H2AB-bait_Rep1_R2.fastq.gz: Figure 9e, f
4Cseq_293T-EAF1mut_HIST2H2AB-bait_Rep2_R1.fastq.gz: Figure 9e, f
4Cseq_293T-EAF1mut_HIST2H2AB-bait_Rep2_R2.fastq.gz: Figure 9e, f
4Cseq_293T-WT_HIST1H2BK-bait_Rep1_R1.fastq.gz: Figure 9c, d
4Cseq_293T-WT_HIST1H2BK-bait_Rep1_R2.fastq.gz: Figure 9c, d
4Cseq_293T-WT_HIST1H2BK-bait_Rep2_R1.fastq.gz: Figure 9c, d
4Cseq_293T-WT_HIST1H2BK-bait_Rep2_R2.fastq.gz: Figure 9c, d
4Cseq_293T-WT_HIST2H2AB-bait_Rep1_R1.fastq.gz: Figure 9e, f
4Cseq_293T-WT_HIST2H2AB-bait_Rep1_R2.fastq.gz: Figure 9e, f
4Cseq_293T-WT_HIST2H2AB-bait_Rep2_R1.fastq.gz: Figure 9e, f
4Cseq_293T-WT_HIST2H2AB-bait_Rep2_R2.fastq.gz: Figure 9e, f
Biotinylation-seq_293T-EAF1mut_Coilin_Input_R1.fastq.gz: Figure 8b-e, h, Figure S13f
Biotinylation-seq_293T-EAF1mut_Coilin_Input_R2.fastq.gz: Figure 8b-e, h, Figure S13f
Biotinylation-seq_293T-EAF1mut_Coilin_pulldown_Rep1_R1.fastq.gz: Figure 8b-e, h, Figure S13f

Biotinylation-seq_293T-EAF1mut_Coilin_pulldown_Rep1_R2.fastq.gz: Figure 8b-e, h, Figure S13f
Biotinylation-seq_293T-EAF1mut_Coilin_pulldown_Rep2_R1.fastq.gz: Figure 8b-e, h, Figure S13f
Biotinylation-seq_293T-EAF1mut_Coilin_pulldown_Rep2_R2.fastq.gz: Figure 8b-e, h, Figure S13f
Biotinylation-seq_293T-EAF1mut_LSM11_Input_R1.fastq.gz: Figure 8f, g
Biotinylation-seq_293T-EAF1mut_LSM11_Input_R2.fastq.gz: Figure 8f, g
Biotinylation-seq_293T-EAF1mut_LSM11_pulldown_Rep1_R1.fastq.gz: Figure 8f, g
Biotinylation-seq_293T-EAF1mut_LSM11_pulldown_Rep1_R2.fastq.gz: Figure 8f, g
Biotinylation-seq_293T-EAF1mut_LSM11_pulldown_Rep2_R1.fastq.gz: Figure 8f, g
Biotinylation-seq_293T-EAF1mut_LSM11_pulldown_Rep2_R2.fastq.gz: Figure 8f, g
Biotinylation-seq_293T-WT_Coilin_Input_R1.fastq.gz: Figure 8b-e, h, Figure S13f
Biotinylation-seq_293T-WT_Coilin_Input_R2.fastq.gz: Figure 8b-e, h, Figure S13f
Biotinylation-seq_293T-WT_Coilin_pulldown_Rep1_R1.fastq.gz: Figure 8b-e, h, Figure S13f
Biotinylation-seq_293T-WT_Coilin_pulldown_Rep1_R2.fastq.gz: Figure 8b-e, h, Figure S13f
Biotinylation-seq_293T-WT_Coilin_pulldown_Rep2_R1.fastq.gz: Figure 8b-e, h, Figure S13f
Biotinylation-seq_293T-WT_Coilin_pulldown_Rep2_R2.fastq.gz: Figure 8b-e, h, Figure S13f
Biotinylation-seq_293T-WT_LSM11_Input_R1.fastq.gz: Figure 8f, g
Biotinylation-seq_293T-WT_LSM11_Input_R2.fastq.gz: Figure 8f, g
Biotinylation-seq_293T-WT_LSM11_pulldown_Rep1_R1.fastq.gz: Figure 8f, g
Biotinylation-seq_293T-WT_LSM11_pulldown_Rep1_R2.fastq.gz: Figure 8f, g
Biotinylation-seq_293T-WT_LSM11_pulldown_Rep2_R1.fastq.gz: Figure 8f, g
Biotinylation-seq_293T-WT_LSM11_pulldown_Rep2_R2.fastq.gz: Figure 8f, g
PROseq_control_Rep1_R1.fastq.gz: Figure 2a-e, Figure S5a-d
PROseq_control_Rep1_R2.fastq.gz: Figure 2a-e, Figure S5a-d
PROseq_control_Rep2_R1.fastq.gz: Figure 2a-e, Figure S5a-d
PROseq_control_Rep2_R2.fastq.gz: Figure 2a-e, Figure S5a-d
PROseq_siCBP80_Rep1_R1.fastq.gz: Figure 2a-e, Figure S5a-d
PROseq_siCBP80_Rep1_R2.fastq.gz: Figure 2a-e, Figure S5a-d
PROseq_siCBP80_Rep2_R1.fastq.gz: Figure 2a-e, Figure S5a-d
PROseq_siCBP80_Rep2_R2.fastq.gz: Figure 2a-e, Figure S5a-d
PROseq_siNELFE_Rep1_R1.fastq.gz: Figure 2a-e, Figure S5a-d
PROseq_siNELFE_Rep1_R2.fastq.gz: Figure 2a-e, Figure S5a-d
PROseq_siNELFE_Rep2_R1.fastq.gz: Figure 2a-e, Figure S5a-d
PROseq_siNELFE_Rep2_R2.fastq.gz: Figure 2a-e, Figure S5a-d
MED26mutPROseq_WT_Rep1_R1.fastq.gz: Figure S7b-k
MED26mutPROseq_WT_Rep1_R2.fastq.gz: Figure S7b-k
MED26mutPROseq_WT_Rep2_R1.fastq.gz: Figure S7b-k
MED26mutPROseq_WT_Rep2_R2.fastq.gz: Figure S7b-k
MED26mutPROseq_MED26mut_Rep1_R1.fastq.gz: Figure S7b-k
MED26mutPROseq_MED26mut_Rep1_R2.fastq.gz: Figure S7b-k
MED26mutPROseq_MED26mut_Rep2_R1.fastq.gz: Figure S7b-k
MED26mutPROseq_MED26mut_Rep2_R2.fastq.gz: Figure S7b-k
amanitinPROseq_NT_Rep1_R1.fastq.gz: Figure S4a-m
amanitinPROseq_NT_Rep1_R2.fastq.gz: Figure S4a-m
amanitinPROseq_NT_Rep2_R1.fastq.gz: Figure S4a-m
amanitinPROseq_NT_Rep2_R2.fastq.gz: Figure S4a-m
amanitinPROseq_amanitin_Rep1_R1.fastq.gz: Figure S4a-m
amanitinPROseq_amanitin_Rep1_R2.fastq.gz: Figure S4a-m
amanitinPROseq_amanitin_Rep2_R1.fastq.gz: Figure S4a-m
amanitinPROseq_amanitin_Rep2_R2.fastq.gz: Figure S4a-m

Field-specific reporting

Please select the one below that is the best fit for your research. If you are not sure, read the appropriate sections before making your selection.

- [x] Life sciences
- [ ] Behavioural & social sciences
- [ ] Ecological, evolutionary & environmental sciences

For a reference copy of the document with all sections, see nature.com/documents/nr-reporting-summary-flat.pdf

Life sciences study design

All studies must disclose on these points even when the disclosure is negative.

Sample size	No statistical methods were used to predetermine sample size. Sample size was determined by pilot experiments. Triplicate samples were used for all of the quantitative analysis except ChIP-seq, PRO-seq, 4C-seq and antibody-based in situ biotinylation-seq. Duplicate samples were used for these analyses. The sample size and number of replicate are described in figure legends.
Data exclusions	There is no data excluded from the analysis.

Replication	We performed experiments at least three times to verify the reproducibility of each experiment except ChIP-seq, PRO-seq, 4C-seq and antibody-based in situ biotinylation-seq. We performed two independent experiments for ChIP-seq, PRO-seq, 4C-seq and antibody-based in situ biotinylation-seq. We confirmed that all attempts were successful.
Randomization	Randomization is not relevant to our research, since we used cell lines and no human or animal samples in this study.
Blinding	High-throughput sequencing was blindly performed, and the data were analyzed based on unbiased analysis method. Other experiments did not require blinding since experimental data obtained through appropriate analysis with normalization method are not affected by human bias.

Reporting for specific materials, systems and methods

We require information from authors about some types of materials, experimental systems and methods used in many studies. Here, indicate whether each material, system or method listed is relevant to your study. If you are not sure if a list item applies to your research, read the appropriate section before selecting a response.

Materials & experimental systems

n/a	Involved in the study
☐	☒ Antibodies
☐	☒ Eukaryotic cell lines
☒	☐ Palaeontology and archaeology
☒	☐ Animals and other organisms
☒	☐ Human research participants
☒	☐ Clinical data
☒	☐ Dual use research of concern

Methods

n/a	Involved in the study
☐	☒ ChIP-seq
☒	☐ Flow cytometry
☒	☐ MRI-based neuroimaging

Antibodies

Antibodies used

Western blotting
The specific antibodies used were as follows: anti-EAF1 antibodies (1:200 dilution, sc-398450; Santa Cruz Biotechnology, Inc., Santa Cruz, CA), anti-MED26 antibodies (D4B1X, 1:1000 dilution, 14950; Cell Signaling Technology, Danvers, MA), anti-ICE1 antibodies (1:1000 dilution, HPA054452; Sigma-Aldrich Corp., St. Louis, MO), anti-ELL antibodies (1:1000 dilution, 14468; Cell Signaling Technology), anti-MED1 antibodies (1:1000 dilution, ab64965; Abcam, Cambridge, UK), anti-MED23 antibodies (1:1000 dilution, A300-425A: Bethyl Laboratories, Montgomery, TX), anti-Rpb1 NTD (1:2000 dilution, 14958; Cell Signaling Technology), anti- phospho-Rpb1-CTD (Ser5) (1:2000 dilution, 13523; Cell Signaling Technology), anti-phospho-Rpb1-CTD (Ser7) (1:2000 dilution, 13780; Cell Signaling Technology), anti-RNA polymerase II-CTD (phospho-2) (1:1000 dilution, ab5095; Abcam), anti-Coilin antibodies (1:2000 dilution, 14168; Cell Signaling Technology), anti-NPAT antibodies (1:300 dilution, sc-136007; Santa Cruz Biotechnology), anti-LSM11 antibodies (1:500 dilution, HPA039587; Sigma-Aldrich Corp.) and anti-β-actin (1:2000 dilution, sc-47778; Santa Cruz Biotechnology, Inc.)

Immunostaining
Primary antibodies to MED26 (1:200 dilution, D4B1X, 14950S; Cell Signaling Technology), MED1 (1:200 dilution, ab64965; Abcam), ELL (1:200 dilution, D7N6U, 14468S; Cell Signaling Technology), ICE1 (1:100 dilution, HPA054452; Sigma-Aldrich Corp.), Coilin (1:2000 dilution, ab11822; Abcam), NPAT (1:200 dilution, HPA066370; Sigma-Aldrich Corp. or sc-136007; Santa Cruz), LSM11 (1:200 dilution, HPA039587; Sigma-Aldrich Corp.), FLASH (1:200 dilution, HPA053573; Sigma-Aldrich Corp.), WRAP53 (1:200 dilution, HPA029928; Sigma-Aldrich Corp.), SMN1 (1:100 dilution, 2F1, 12976S; Cell Signaling Technology) or CDK11 (1:200 dilution, HPA073626; Sigma-Aldrich Corp.) were used in PBST containing 10% BSA and 10% horse serum. Alexa Fluor 488-labelled goat polyclonal antibody to rabbit IgG (H+L) (1:2000 dilution, A-11034, Life Technologies, Carlsbad, CA) or Alexa Fluor 555-labelled goat polyclonal antibody to mouse IgG1 (1:2000 dilution, A-21424, Life Technologies) were used in PBS..

ChIP
The specific antibodies used were as follows: anti-ELL (14468; Cell Signaling Technology), anti-RPB1 NTD (D8L4Y, 14958; Cell Signaling Technology), anti-phospho-RPB1 CTD (Ser2) (ab5095; Abcam), anti-phospho-RPB1 CTD (Ser5) (D9N5I, 13523; Cell Signaling Technology), anti-phospho-RPB1 CTD (Ser7) (E2B6W, 13780; Cell Signaling Technology) and normal rabbit IgG antibodies (PM035; MBL).

Antibody-based in situ biotinylation
The specific antibodies used were as follows: anti-Coilin (ab11822; Abcam), anti-LSM11 (HPA039587; Sigma-Aldrich Corp.)

Validation

MED26 (D4B1X, 14950S; Cell Signaling Technology): https://en.cellsignal.jp/products/primary-antibodies/med26-d4b1x-rabbit-mab/14950
Validated for western blotting (WB), immunoprecipitation (IP) and chromatin immunoprecipitation (ChIP) in HeLa cell lysate.

MED1 (ab64965; Abcam): https://www.abcam.com/trap220med1-antibody-ab64965.html
Validated for WB in human Jurkat cells and U2OS cells and immunohistochemistry (IHC) in formalin/parafolmaldehide (PFA)-fixed human breast carcinoma tissue.

ELL (D7N6U, 14468S; Cell Signaling Technology): https://en.cellsignal.jp/products/primary-antibodies/ell-d7n6u-rabbit-mab/14468?site-search-type=Products&N=4294956287&Ntt=ell&fromPage=plp
Validated for WB, IP, immunofluorescence (IF) and ChIP in human cultured cell lines.
.
ICE1 (HPA054452; Sigma-Aldrich Corp.): https://www.sigmaaldrich.com/JP/ja/product/sigma/hpa054452, https://www.proteinatlas.org/ENSG00000164151-ICE1/antibody#western_blot
We confirmed the antibody specifically detect signal at predicted size in WB and nuclear/nuclear body staining in IF.

Coilin (ab11822; Abcam): https://www.abcam.com/coilin-antibody-pdelta-ab11822.html
Abcam stated that the antibody is tested and suitable for WB and IF.

NPAT (HPA066370; Sigma-Aldrich Corp.): https://www.sigmaaldrich.com/JP/ja/product/sigma/hpa066370, https://www.proteinatlas.org/ENSG00000149308-NPAT/antibody
Validated for IF in PFA-fixed human cells.
Human Protein Atlas provides images for IF in human CACO-2 cells, SK-MEL-30 cells, and U2OS cells.

NPAT (sc-136007; Santa Cruz)
Santa Cruz provided images for WB in Jurkat cell lysate and IF in A-431 cells. This product is no longer available.

LSM11 (HPA039587; Sigma-Aldrich Corp.): https://www.sigmaaldrich.com/JP/ja/product/sigma/hpa039587, https://www.proteinatlas.org/ENSG00000155858-LSM11/antibody
Validated for IF in PFA-fixed human cells and IHC in human stomach.
Human Protein Atlas provides images for IF in human A-431 cells, U2OS cells, and U-251MG cells.

FLASH (HPA053573; Sigma-Aldrich Corp.): https://www.sigmaaldrich.com/JP/ja/product/sigma/hpa053573, https://www.proteinatlas.org/ENSG00000118412-CASP8AP2/antibody
Validated for IF in PFA-fixed human cells.
Human Protein Atlas provides images for IF in human HEK293 cells, RH-30 cells, and U2OS cells.

WRAP53 (HPA029928; Sigma-Aldrich Corp.): https://www.sigmaaldrich.com/JP/ja/product/sigma/hpa029928, https://www.proteinatlas.org/ENSG00000141499-WRAP53/antibody
Validated for IF in PFA-fixed human cells and WB in human cell lysate.
Human Protein Atlas provides images for IF in human A-431 cells, U2OS cells, and U-251MG cells.

SMN1 (2F1, 12976S; Cell Signaling Technology): https://en.cellsignal.jp/products/primary-antibodies/smn1-2f1-mouse-mab/12976?site-search-type=Products&N=4294956287&Ntt=12976s&fromPage=plp&_requestid=5906896
Validated for WB, IP and IF in human cultured cell lines.

CDK11 (HPA073626; Sigma-Aldrich Corp.): https://www.sigmaaldrich.com/JP/ja/product/sigma/hpa073626, https://www.proteinatlas.org/ENSG00000248333-CDK11B/antibody
Validated for IF in PFA-fixed human cells.
Human Protein Atlas provides images for IF in human SK-MEL-30 cells, SiHa cells, and U2OS cells.

Eukaryotic cell lines

Policy information about cell lines

Cell line source(s)	Human embryonic kidney 293T (HEK293T) cell line, HeLa cell line, HCT116 cell line.<br>Drosophila Schneider 2 (S2) cell line: provided by Dr. Kuniaki Saito (National Institute of Genetics).
Authentication	Because these cell lines has been recurrently and generally used by many researchers in previous works, we have not done further authentication about them.
Mycoplasma contamination	We confirmed that all of the cell lines were negative for mycoplasma contamination.
Commonly misidentified lines<br>(See ICLAC register)	There is no commonly misidentified cell lines in this research.

ChIP-seq

Data deposition

☒ Confirm that both raw and final processed data have been deposited in a public database such as GEO.

☒ Confirm that you have deposited or provided access to graph files (e.g. BED files) for the called peaks.

Data access links<br>*May remain private before publication.*	GEO accession code: GSE164144<br>Please go to https://www.ncbi.nlm.nih.gov/geo/query/acc.cgi?acc=GSE164144 and enter token "ixkxomaofncdxkl" into the box.
Files in database submission	ChIPseq_293T-WT_ELL_Input_R1.fastq.gz

	ChIPseq_293T-WT_ELL_Input_R2.fastq.gz<br>ChIPseq_293T-WT_ELL_IP_Rep1_R1.fastq.gz<br>ChIPseq_293T-WT_ELL_IP_Rep1_R2.fastq.gz<br>ChIPseq_293T-WT_ELL_IP_Rep2_R1.fastq.gz<br>ChIPseq_293T-WT_ELL_IP_Rep2_R2.fastq.gz
Genome browser session (e.g. UCSC)	no longer applicable

Methodology

Replicates	In each experiment, ChIP-seq was performed in duplicate.
Sequencing depth	ChIPseq_293T-WT_ELL_Input: Total number of reads 23,877,655, uniquely mapped reads 22,931,606, 100 paired-end<br>ChIPseq_293T-WT_ELL_IP_Rep1: Total number of reads 17,946,946, uniquely mapped reads 17,418,398, 100 paired-end<br>ChIPseq_293T-WT_ELL_IP_Rep2_R1: Total number of reads 18,327,829, uniquely mapped reads 17,793,691, 100 paired-end
Antibodies	anti-ELL (14468; Cell Signaling Technology)
Peak calling parameters	Peak calling was not performed in this study.
Data quality	All of the ChIP-seq samples have more than 95% unique mapped reads to the human genome.
Software	Adapter sequences were trimmed using Trim Galore (v 0.64_dev), and reads were mapped to human genome GRCH38 using the Bowtie2 alignment tool (version 2.3.5.1) with the default settings.
